# Inhibitory Potential against Digestive Enzymes Linked to Obesity and Type 2 Diabetes and Content of Bioactive Compounds in 20 Cultivars of the Peach Fruit Grown in Poland

**DOI:** 10.1007/s11130-018-0688-8

**Published:** 2018-10-03

**Authors:** Paulina Nowicka, Aneta Wojdyło, Piotr Laskowski

**Affiliations:** 1 Department of Fruit, Vegetable and Plant Nutraceuticals Technology, Wrocław University of Environmental and Life Sciences, 37 Chełmońskiego Street, 51-630 Wroclaw, Poland; 2 Research Station for Cultivar Testing in Zybiszów near Wrocław, 55-080 Kąty Wrocławskie, Poland

**Keywords:** Peach fruit, Bioactive compounds, Antioxidant capacity, Antidiabetic activity

## Abstract

The presented study provides important insights on the health properties of *Prunus persica* fruit related to their polyphenol and carotenoid profiles, antioxidant capacity and *in vitro* potential to inhibit enzymes relevant to type 2 diabetes (α-amylase, α-glucosidase) and obesity (pancreatic lipase) management. Such results have not been published so far. The study showed substantial differences in the chemical composition of peach fruit depending on the cultivar. At the same time, it demonstrated some common features of selected cultivars - the varieties with light flesh (‘Spring Time’; ‘Madison’) were characterized by a high content of phenolic acids and flavonols, thus exhibiting high activity against α-amylase, while the yellow varieties with high content of carotenoids (‘Harrow Diamond’; ‘Harrow Beauty’) showed high inhibitory activity toward porcine pancreatic lipase. Finally, it has been shown that peach fruit is an interesting raw material with a varied chemical composition and nutritional value, especially with high inhibitory potential against digestive enzymes linked to obesity and type 2 diabetes, strongly determined by the cultivar.

## Introduction

Peach (*Prunus persica* L. Batsch) belongs to the *Rosaceae* family and is an initial species for many cultivars commonly grown worldwide. *Prunus persica* fruit is a stone fruit suitable for direct consumption and an interesting material for processing. For several years it has arisen a growing interest among consumers and food processors not only due to its attractive sensory values but also its chemical composition and health-promoting properties which are still sparsely analyzed and scientifically documented. Therapeutic properties of peach fruit, stones, flowers, and leaves have been exploited for years, especially in the folk medicine in China – the country of origin of this fruit material. *P. persica* fruits have been ascribed many health-promoting properties in diseases of the cardiac muscle, kidneys, liver and gallbladder, peach flowers – in diseases of the stomach and in rheumatism, whereas peach stones were demonstrated to exhibit antimycotic and bactericidal properties, and leaves – to have a positive effect on a woman’s body [1].

The basic chemical components of peach fruit include: carbohydrates, acids, vitamins (C, PP and B group), and minerals (magnesium, phosphorus, iron, boron, potassium, folic acid, calcium). In addition, fruits of this species contain pectins and carotenoids that impart the characteristic orange color to their skin. Noteworthy are also their other biologically-active compounds like, *e.g.*, polyphenols, however investigations conducted thus far have mainly been focused on fatty acid analysis of this species, neglecting other phytochemicals that are also significant from the viewpoint of health-promoting properties [1]. Little known polyphenolic fractions of peach fruit are derivatives from the group of flavonols and flavan-3-ols, including procyanidin polymers. Despite difficulties in their analysis, they have been demonstrated to be one of the key plant metabolites owing to a wide spectrum of their health-promoting properties. Phenolic compounds constitute a very numerous group of natural organic substances that occur in various morphological parts of plants. They exhibit especially strong antioxidative properties that protect defense systems of the body against destructive effects of free radicals. Therefore, their intake prevents the disruption of the antioxidative equilibrium and - by this means - prevents the incidence of many disease entities, *i.e.*, neoplasmic diseases, cardiovascular diseases, diabetes or neurodegenerative diseases [2]. Many studies have also proved the consumption of food products rich in these components to cause reduction in blood level of glucose, to contribute to the inhibition of the atherosclerotic process and to improve eye functions [3]. Little literature data is however available regarding the profiles and concentrations of organic acids or sugar profile in fruits of peach [4, 5].

In addition, previous studies on the peach fruit often investigated chemical, and nutritional properties of the fruit, but usually of one selected cultivar. No scientific research combining both phenolic characterization, qualitative chemical profile, antioxidant and inhibitory potential against digestive enzymes linked to obesity and type 2 diabetes analyses in different peach cultivars grown in Poland has been carried out so far.

Therefore, the present study evaluated fruits of 20 different cultivars of peach for their bioactive compounds (phenolic compounds and carotenoids), antioxidant capacities, potential to inhibit enzymes relevant to hyperglycemia (α-amylase, α-glucosidase) and obesity (pancreatic lipase) management. The additional aim of this study was to find the most promising peach cultivars for the Polish food industry and for direct consumption considering their quality, polyphenolic constituents, and high nutrient levels. No cultivars of the peach fruit have been analyzed from this perspective until now.

## Materials and Methods

### Plant Material

Twenty cultivars of peach fruit (*Prunus persica*): ‘Harbinger’, ‘Kijowska Wczesna’, ‘Spring Time’, ‘Beta’, ‘Maycresh’, ‘Harrow Diamond’, ‘Dixired’, ‘Candor’, ‘Harnaś’, ‘Sweet Haven’, ‘WB 258’, ‘Early Redhaven’, ‘SB6A–35’, ‘Jerseyland’, ‘BL6’, ‘Red Cup’, ‘Royalvee’, ‘Flamin Fury’, ‘Harrow Beauty’, ‘Madison’ were used in this study. All of them were appropriate for food manufacturing and grown in Poland. The fruit obtained from 8 year-old trees were hand-harvested and were collected at “ready-to-eat” ripening stage (the degree of ripeness was determined on the basis of separability and fruit coloring) in July and August 2016. The peaches were harvested at the Research Station for Cultivar Testing in Zybiszów near Wrocław (Poland) (51° 3′ 51.11″N, 16°54′43.56″ E). In the course of the measurements, 6 replications (6 randomly chosen fruits) from 3 trees, that is, 18 replications *per* variety, were established. For the polyphenolic compounds, and pro-health properties analyses the whole fruits were frozen by liquid nitrogen and crushed to homogeneous powder in laboratory mill and after that freeze-dried and stored in a freezer (−80 °C; Frilabo; Lyon, France) until analysis.

### Analysis of Polyphenol Compounds

The extracts for the quantitative analysis of polyphenols by UPLC were performed as described by Wojdyło et al. [6]. In turn the analysis of polymeric procyanidins by phloroglucinol method was performed according to the protocol described by Kennedy and Jones [7]. The results were expressed as mg/100 g dm of peach.

### Analysis of Carotenoids

For the extraction and determination of carotenoids by UPLC a protocol described earlier by Wojdyło et al. [8] was followed. The results were expressed as mg/100 g dm of peach.

### Analysis of Antioxidant Capacity by ABTS and FRAP Methods

A solvent for the antioxidant capacity assays were prepared according to Wojdyło et al. [6]. The ABTS°^+^ and FRAP analysis were determined as previously described by Re et al. [9] and Benzie and Strain [10], respectively. Results were expressed as mmol Trolox/100 g dm of peach.

### α-Amylase, α-Glucosidase and Pancreatic Lipase Inhibition Assays

The α-amylase and α-glucosidase inhibitory effect of the peach kernel extracts was assayed according to the procedure described previously by Nowicka et al. [11] while the inhibition of lipase activity was determined according to Podsędek et al. [12], respectively. The acarbose was included in the case of α-amylase and α-glucosidase as a positive control, while the Orlistat was used as a positive control for pancreatic lipase. The results were expressed as IC_50_ value.

### Statistical Analysis

Statistical analysis was conducted using Statistica version 12.50 (StatSoft, Krakow, Poland). Significant differences (*p* ≤ 0.05) between means were evaluated by one-way ANOVA and Duncan’s multiple range test. All data included in this study are presented as the mean value±standard deviation and were performed at least three times.

## Result and Discussion

### Quantification of Polyphenols in Different Cultivars of Peaches

Table 1 presents results of determinations of polyphenols content in fruits of the studied cultivars of peaches. Four groups of phenolic compounds were identified in each analyzed cultivar, *i.e.*: phenolic acids, flavonols, anthocyanins, and flavan-3-ols (monomers, dimers, and polymeric procyanidins).

**Table 1:** Content of phenolic compounds and carotenoids [mg/100 g dm] in different peach cultivars

Cultivars	Antocyanins	Phenolic acid	Flavonols	Flavan-3-ols (monomers and dimers)	Polymeric procyanidins	Total polyphenols	Total carotenoids
Harbinger	55.60 ± 0.05 c^ǂ^	207.09 ± 3.87 f	33.24 ± 1.04 a	20.18 ± 0.04 l	1192.27 ± 31.77 i	1508.38 ± 36.77 k	392.50 ± 11.02 a
Kijowska Wczesna	29.97 ± 0.03 h	221.13 ± 4.13 e	22.16 ± 0.05 g	nd	2406.92 ± 42.21 b	2680.18 ± 46.42 b	76.00 ± 9.07 i
Spring Time	74.48 ± 0.60 a	86.57 ± 1.63 m	27.09 ± 0.12 c	18.20 ± 0.01 m	1025.12 ± 30.75 jk	1231.46 ± 33.11 m	40.08 ± 5.12 j
Beta	21.86 ± 0.20 k	62.67 ± 1.18 pr	19.56 ± 0.37 ij	27.34 ± 0.00 j	1556.64 ± 26.70 f	1688.07 ± 28.45 i	290.34 ± 17.21 c
Maycresh	58.85 ± 0.50 b	57.31 ± 1.05 r	26.66 ± 0.41 cd	18.36 ± 0.14 m	991.78 ± 19.75 k	1152.96 ± 21.85 n	203.82 ± 8.24 g
Harrow Diamond	56.00 ± 0.57 c	192.12 ± 3.54 g	26.16 ± 0.11 d	51.24 ± 0.11 g	1821.01 ± 24.63 d	2146.53 ± 28.96 e	253.03 ± 2.65 e
Dixired	39.12 ± 0.34 d	66.62 ± 1.24 op	25.23 ± 0.08 e	33.18 ± 0.06 i	1599.69 ± 32.99 f	1763.84 ± 34.71 h	243.52 ± 11.87 ef
Candor	35.21 ± 2.21 f	121.07 ± 2.21 k	30.01 ± 0.87 b	94.84 ± 1.09 a	2175.90 ± 25.27 c	2457.03 ± 31.65 d	245.36 ± 6.29 ef
Harnaś	10.81 ± 0.68 l	234.08 ± 4.31 d	17.48 ± 0.27 k	53.96 ± 0.21 f	1674.66 ± 40.24 e	1990.99 ± 45.71 g	219.34 ± 10.03 g
Sweet Haven	38.22 ± 0.03 de	92.25 ± 1.73 l	15.59 ± 0.00 l	11.63 ± 0.21 o	832.69 ± 14.98 l	990.38 ± 16.958 o	214.46 ± 1.68 g
WB 258	25.41 ± 1.09 j	185.46 ± 3.46 h	21.29 ± 0.04 h	64.54 ± 0.11 c	2819.57 ± 54.58 a	3116.27 ± 59.28 a	280.54 ± 12.95 cd
Early Redhaven	27.92 ± 0.20 i	139.92 ± 2.54 j	19.18 ± 0.62 j	16.89 ± 0.01 n	1366.36 ± 20.99 g	1570.27 ± 24.36 j	228.88 ± 5.87 f
SB6A - 35	28.88 ± 0.02 hi	266.12 ± 4.92 c	21.87 ± 0.74 gh	55.35 ± 0.00 e	1719.82 ± 21.59 e	2092.04 ± 27.27 f	354.95 ± 11.11 b
Jerseyland	26.22 ± 1.04 j	305.75 ± 5.61 a	13.57 ± 0.01 m	83.29 ± 1.41 b	2195.56 ± 35.87 c	2624.39 ± 43.94 c	379.46 ± 8.63 ab
BL6	30.01 ± 0.03 h	194.28 ± 3.58 g	23.37 ± 0.11 f	58.70 ± 0.69 d	1847.40 ± 25.42 d	2153.76 ± 29.83 e	342.13 ± 5.04 b
Red Cup	37.55 ± 0.35 e	70.30 ± 1.32 o	22.88 ± 0.00 f	17.28 ± 0.55 n	585.10 ± 9.55 m	733.11 ± 11.77 p	279.88 ± 10.11 d
Royalvee	32.95 ± 1.07 g	159.38 ± 2.93 i	20.14 ± 0.61 i	35.23 ± 0.00 h	1042.10 ± 21.26 j	1289.80 ± 25.87 l	293.98 ± 5.32 c
Flamin Fury	36.01 ± 0.26 f	80.61 ± 1.51 n	18.04 ± 0.07 k	21.90 ± 0.14 k	985.39 ± 19.56 k	1141.95 ± 21.54 n	61.79 ± 4.78 i
Harrow Beauty	6.59 ± 0.01 m	273.32 ± 5.07 b	14.98 ± 0.03 l	55.92 ± 0.80 e	1250.83 ± 17.52 h	1601.64 ± 23.43 j	257.15 ± 5.81 e
Madison	3.10 ± 0.09 n	209.54 ± 3.88 f	10.11 ± 0.21 n	11.98 ± 0.21 o	487.85 ± 4.64 n	722.58 ± 9.03 p	174.17 ± 4.02 h
minimum	3.10	57.31	10.11	11.64	487.85	722.58	40.08
maximum	74.48	305.75	33.24	94.84	2819.57	3116.27	392.50
mean	33.74	161.28	21.43	37.50	1478.83	1732.78	241.57

^ǂǂ^value±SD are means of three repetitions

^ǂ^Mean values followed by different letters are statistically different at *p* < 0.05

The total content of polyphenols differed significantly among the analyzed peach cultivars and ranged from 722.58 mg/100 g dm in ‘Madison’ to 3116.27 mg/100 g dm in ‘WB 258’, while the average value was 1732.38 mg/100 g dm of peaches. According to other authors, the wide range of polyphenol contents may be attributed to different species, geographical origin, climatic factors and cultivation conditions [13]. Other authors showed also that the late-maturing cultivars had a higher content of total polyphenolic compounds than the early-maturing cultivars [13], but such observations were not confirmed in our study.

The main fraction of polyphenolic compounds determined in the peaches were flavan-3-ols, with the majority being of polymeric procyanidins. Tomás-Barberán et al. [5] also reported the presence of procyanidin dimers of the B- and A-types, as well as the procyanidin trimers in peaches with different color of the pulp. In addition, Mokrani et al. [14] demonstrated that procyanidins were the main class of phenolic compounds accounting for an average percentage higher than 53% of all phenolics, which is consistent with results obtained in our research. The phloroglucinol method was employed in this study to determine the content of procyanidins in peach fruits. Generally, the content of polymeric procyanidins ranged from 487.85 mg/100 g dm (‘Madison’) to 2819.57 mg/100 g dm (‘WB 258’). The analysis shows that the main factor determining the content of polymeric flavan-3-ols in peaches is their cultivar.

The second fraction of phenolics was represented by phenolic acids (from 57.31 mg/100 g dm in ‘Maycresh’ to 305.75 mg/100 g dm in the ‘Jerseyland’). They accounted for an average around 10% of total polyphenolic compounds. Tangible differences were observed in the phenolic acid between the white flesh (‘Spring Time’; ‘Early Redhaven’; ‘Red Cup’ or ‘Flamin Fury’) and orange/yellow flesh (‘Harrow Diamond’, ‘Harnaś’, ‘WB 258’ or ‘Jerseyland’) cultivars. The peaches with lighter pulp had a lower content of phenolic acids than these with yellow flesh. In turn, there was no clear trend in phenolic acids content with the ripening of the different cultivars – this tendency was also earlier described by other authors [5, 14, 15]. Many authors have confirmed a high content of phenolic acids in peaches, pointing also that the main compounds in this group are chlorogenic and neochlorogenic acids [5, 15]. The content of phenolic acids, especially of chlorogenic acid, is very important because they have been identified as the main phenolic compounds responsible for the *in vitro* anti-cancer (breast, lung, colon and liver) activities. In addition, the peach fruit contain quinic acid derivatives, which may help alleviate urinary tract infection.

Another group of polyphenols identified in the analyzed peach fruits were flavonols. For different cultivars of peaches, the total flavonols content ranged from 10.11 mg/100 g dm in ‘Madison’ cv. to 33.24 mg/100 g dm in ‘Harbringer’ cv., with the average value of 21.43 mg *per* 100 g of dried peaches. It was observed that the early-maturing cultivars, such as: ‘Kijowska Wczesna’, ‘Harbringer’ and ‘Spring Time’, had higher contents of flavonols than the late-maturing ones (‘Madison’, ‘Flamin Fury’, ‘Harrow Beauty’). Other authors showed no relationship between the content of flavonols and cultivar type (late- or early-maturing), but they observed that the flavonols content decreases as the fruit expanded and matured and also that during storage, especially with access to light, the content of flavonols decreased drastically [16, 17].

The studied cultivars of peaches were also demonstrated to differ significantly in anthocyanin content. The final content of anthocyanins largely defined the color of the skin, therefore the cultivars with intense red skin were characterized by a higher content of anthocyanins than these with the light peel. The anthocyanin content ranged from 3.10 mg/100 g dm in ‘Madison’ fruit to 74.48 mg/100 g dm in ‘Spring Time’ fruit, with the average content accounting for 33.74 mg/100 g dm of peaches. Literature data show that anthocyanins constitute a small group of compounds in peach fruits and only in some cultivars [14, 15]. Both anthocyanin content and their composition in fruit depend on environmental factors, as well as on postharvest processing conditions [14, 16].

### Quantification of Carotenoids in Different Cultivars of Peaches

In the presented study also carotenoids content was determined in different cultivars of peach fruits (Table 1). Carotenoids are natural pigments involved in the photosynthetic process, which play indispensable roles in providing pigmentation for fruit [18]. The peach fruits are considered to be one of the richest sources of these compounds among the fruit, next to the apricots, persimmon or mango [1]. In the analyzed fruit, the content of carotenoids ranged from 40.08 mg/100 g dm in the case of the early-maturing cultivar with light flesh – ‘Spring Time’ to 392.50 mg/100 g dm also in the early-maturing cultivar, but with yellow pulp – ‘Harbringer’. Among the twenty tested cultivars of peaches, twelve were characterized by the carotenoids content between 200 and 300 mg/100 g dm, four of them had below of 200 mg of these plant pigments/100 g dm, and the other four had above 300 mg carotenoids/100 g dm of peaches. This study showed that the main factor determining the content of carotenoids in peach fruits is their cultivar. These observations are consistent with findings of other authors who showed additionally that the content of carotenoids increased successively during fruit development in the case of cultivars with yellow flash, but remained stable in the peach fruits with light flash during the whole process of fruit development [15, 18].

### Antioxidative Capacity in Peach Fruit

Two different *in vitro* assays: ABTS and FRAP were used to evaluate the antioxidative capacity of the twenty studied peaches cultivars (Table 2). Significant differences were observed among the cultivars with reference to the antioxidative potential, but the fruit peach extracts displayed similar antioxidative capacities when assayed with both the methods applied (PC = 0.987). The highest antioxidative capacity was found for ‘WB-258’ cultivar which was also characterized by the highest content of polyphenols, while the lowest antioxidative capacity was displayed by ‘Red Cup’ – a late-maturing cultivar with a low content of polyphenols. It was observed that not only the total content of polyphenols but also the type of phenolic compounds played a very important role in the antioxidative capacity of peach fruit. The obtained results suggest that the antioxidative capacity of the peach fruit is related to the presence of total polyphenols, especially flavan-3-ols and can be ascribed to phenolic acids. Positive correlations were found between the results of both total antioxidative assays and total phenolics content (PC = 0.903 and 0.913 for ABTS and FRAP, respectively), between the antioxidative capacity and flavan-3-ols content (PC = 0.879 and 0.887 for ABTS and FRAP, respectively) and also between the antioxidative capacity and phenolic acids content (PC = 0.689 and 0.691 for ABTS and FRAP, respectively). It means that phenolic compounds present in these fruit are the major contributors to the reducing power and scavenging radical capacities. It may, therefore, be speculated that the major role of peach fruit polyphenols is that they act as potent radical scavengers and primary chain-breaking antioxidants, what was confirmed by other authors [19].

**Table 2:** Enzyme of α-amylase. α-glucosidase, pancreatic lipase inhibitory activities and antioxidant capacity of analyzed peach

Cultivars	Antioxidant capacity [mmol Trolox/100 g dm]		Enzyme inhibition IC_50_ [mg of dried fruit]		
	ABTS	FRAP	α-amylase	α-glucosidase	pancreatic lipase
Harbinger	2.99 ± 0.31 ef^ǂ^	2.15 ± 0.08 ij	64.85 ± 0.23 h	144.00 ± 1.00 c	1.39 ± 0.18 b
Kijowska Wczesna	6.07 ± 0.18 ab	3.82 ± 0.08 b	33.10 ± 0.12 n	26.40 ± 0.80 k	1.10 ± 0.01 c
Spring Time	2.69 ± 0.06 f	2.06 ± 0.12 j	35.65 ± 0.59 lm	81.20 ± 0.80 f	1.44 ± 0.10 b
Beta	3.15 ± 0.16 ef	2.12 ± 0.06 ij	44.01 ± 0.41 k	63.40 ± 0.60 h	1.30 ± 0.00 bc
Maycresh	2.44 ± 0.14 f	1.52 ± 0.03 kl	35.12 ± 0.00 m	133.40 ± 1.00 d	1.06 ± 0.16 c
Harrow Diamond	4.21 ± 0.58 e	3.02 ± 0.04 fg	33.71 ± 0.47 mn	26.80 ± 0.20 k	0.07 ± 0.00 h
Dixired	3.67 ± 0.43 e	2.28 ± 0.08 hi	45.52 ± 1.63 k	214.40 ± 1.60 a	2.06 ± 0.05 a
Candor	5.26 ± 0.13 bcd	3.42 ± 0.12 cd	73.72 ± 2.92 f	28.80 ± 0.20 k	1.53 ± 0.06 b
Harnaś	5.02 ± 0.23 de	3.17 ± 0.09 ef	52.88 ± 0.38 j	29.60 ± 0.20 k	0.33 ± 0.05 f
Sweet Haven	2.49 ± 0.14 f	1.65 ± 0.06 k	37.10 ± 0.84 l	155.40 ± 1.40 b	0.25 ± 0.00 g
WB 258	6.65 ± 0.10 a	4.45 ± 0.19 a	58.42 ± 0.27 i	26.20 ± 0.20 k	0.59 ± 0.00 f
Early Redhaven	3.60 ± 4.25 e	2.37 ± 0.08 h	63.12 ± 1.23 h	93.00 ± 1.00 e	1.53 ± 0.00 b
SB6A - 35	5.31 ± 0.44 bc	3.15 ± 0.26 ef	91.08 ± 0.00 c	43.80 ± 0.40 j	0.51 ± 0.11 f
Jerseyland	5.31 ± 0.09 bc	3.51 ± 0.05 c	84.16 ± 1.54 d	27.20 ± 0.00 k	0.46 ± 0.01 f
BL6	4.27 ± 0.18 e	2.89 ± 0.01 g	110.78 ± 2.77 b	29.00 ± 4.20 k	0.88 ± 0.00 d
Red Cup	2.04 ± 0.03 g	1.38 ± 0.06 l	91.09 ± 0.90 c	210.20 ± 3.20 a	0.49 ± 0.02 f
Royalvee	3.01 ± 0.35 ef	2.10 ± 0.02 ij	66.77 ± 0.03 g	72.00 ± 0.60 g	1.26 ± 0.02 c
Flamin Fury	2.39 ± 0.09 f	1.70 ± 0.02 k	141.39 ± 0.04 a	42.60 ± 0.40 j	0.64 ± 0.00 e
Harrow Beauty	4.99 ± 0.52 de	3.26 ± 0.22 de	77.94 ± 0.10 e	25.20 ± 1.4 k	0.38 ± 0.08 f
Madison	3.41 ± 0.13 e	2.31 ± 0.19 hi	28.26 ± 0.05 o	50.20 ± 0.40 i	0.25 ± 0.02 g

value±SD are means of three repetitions

^ǂ^Mean values followed by different letters are statistically different at *p* < 0.05

Definitely less positive correlations, than in the case of phenolic compounds, were found between the antioxidative capacity and content of carotenoids (PC = 0.197 and 0.185 for ABTS and FRAP, respectively). Many researchers indicate that the structure of conjugated double bonds in the polyene backbone influences the high antioxidative capacity of carotenoids [14, 19], but such a high impact of carotenoids on the final antioxidative potential was not observed in our study. However, it may be speculated that although carotenoid compounds do not contribute so effectively to the hydrogen and electron donating abilities, they might be involved in the chelating potency, which also suggested other authors [14].

### Inhibitory Activities of Peach toward Digestive Enzymes

In this work, the inhibition of α-glucosidase, α-amylase, and pancreatic lipase by 20 cultivars of peach extracts were investigated and presented as IC_50_ values in Table 2. Pancreatic lipase is a key enzyme in dietary fat absorption, responsible for the hydrolysis of 50–70% of dietary triglycerides into monoacylglycerides and free fatty acids, which can then be absorbed by enterocytes. Inhibition of this enzyme is used to reduce dietary fat absorption and therefore the use of phytoextracts as pancreatic lipase inhibitors may represent an alternative approach for weight loss treatment [20]. Among the peach cultivars with reference to the inhibitory activity toward porcine pancreatic lipase, significant differences (*p* < 0.05) were observed. The inhibitory effect of the peach fruit ranged from 0.07 mg/ml (‘Harrow Diamond’) to 2.06 mg/ml (‘Dixired’). It was observed that the late-maturing cultivars were characterized by the most active (‘Madison’ > > ‘Harrow Beauty’ and ‘Flaming fury’), while the early-maturing peaches showed the highest IC_50_ value (more than 1 mg/ml). Generally, it was not observed positive relationship between inhibitory activity toward porcine pancreatic and the total content of polyphenols, only phenolic acid showed so correlation (PC = 0.457). In turn, high positive relationship was observed between pectins content and inhibitory activity against lipase (PC = 0.538 and 0.691, respectively). It is well known that pectins inhibit the absorption of dietary fats and their deposition in liver tissues as well as reduce glucose concentration in blood. Furthermore, when coupled with antioxidative compounds, they inhibit proteins of tyrosine phosphatase that are a negative regulator of insulin signaling [21, 22].

The incidence of obesity is associated with the incidence of type 2 diabetes, characterized by elevated blood glucose levels. Type 2 diabetes represents 90% of all diabetes cases and among other diet related diseases is the major cause of deaths. The insulin-independent diabetes is linked with insulin resistance which precedes, or the dysfunction of β cells that are no longer capable of synthesizing a sufficient quantity of insulin to maintain normoglycemia caused [22]. It was showed that the polyphenols have many biological activities and constitute an important part of the human diet also during the prevention of type 2 diabetes. Their hypoglycemic action results from their antioxidative potential involved in restoring insulin secreting machinery in pancreatic cells, or abilities to inhibit the activity of carbohydrates hydrolyzing enzymes (α-amylase and α-glucosidase) [12, 22]. The significant differences (*p* < 0.05) were found among the analyzed samples in the inhibitory activities toward α-amylase, and α-glucosidase. The inhibition of α-amylase, presented as the IC_50_ values, ranged from 28.26 to 141.39 mg/ml, while of α-glucosidase were from 25.20 to 214.40 mg/ml. The inhibitory effect toward α-glucosidase stimulated the most the content of polyphenols (PC = 0.564), but the highest positive correlation was observed in the case of phenolic acid (PC = 0.609) and the content of polimeric procyanidins (PC = 0.525). Other study suggested that flavonols can interact with hydroxycinnamic acids increasing the inhibition of α-glucosidase [23]. In turn, α-amylase showed the positive relationship with anthocyanins content (PC = 0.356), but generally the peaches are better inhibitors of lipase and α-glucosidase, than α-amylase. Despite the promising results obtained in this study, further research is required to confirm that plant components can inhibit lipase, α-amylase and α-glucosidase *in vivo*, prevent fat intake and carbohydrates absorption and do so at levels achievable from normal diets.

## Conclusion

The presented study provides important insights on the health properties of *Prunus persica* fruit growing in Poland related to their polyphenol and carotenoid profiles, antioxidant capacity and *in vitro* potential to inhibit enzymes relevant to hyperglycemia (α-amylase, α-glucosidase) and obesity (pancreatic lipase) management. Such results have not been published so far.

It has been shown that the common feature for the ‘Jerseyland’, ‘Candor’, ‘Kijowska Wczesna’, ‘Harnaś’, ‘WB 258’, ‘SB6A–35’, ‘BL6’ cvs. were the high content of polyphenolic compounds, including flavan-3-ols and phenolic acids, and also antioxidative capacity and inhibitory potential against α-glucosidase being the highest among all analyzed peach cultivars. In addition, the obtained results showed that the early-maturing varieties as ‘Kijowska Wczesna’, ‘Harbringer’, ‘Spring Time’, ‘Dixired’, ‘Maycresh’, ‘Harrow Diamond’, and ‘Early Redhaven’ were characterized by a high content of anthocyanins and flavonols. A high antioxidative potential of the tested raw material was demonstrated, but also its ability to inhibit enzymes linked to obesity and type 2 diabetes. It was indicated that the compounds contained in peaches were characterized by several times higher health-promoting activity than that determined in apples or red currant. Our study showed also that the health-promoting properties of peaches are stimulated mainly by polymeric procyanidins, pectins and hydroxycinnamic acids.

Finally, it has been shown that peach fruit is an interesting raw material with high pro-health properties potential, strongly determined by the cultivar. However, further *in vitro* and *in vivo* studies are necessary to confirm a complex interaction between phytochemicals, which produce synergistic responses and also to confirm the nutritional value of peach fruits, especially for the prevention of obesity and type 2 diabetes.

## Abbreviations

ABTS: 2,2′-Azino-bis(3-ethylbenzothiazoline-6-sulphonic acid)
dm: dry matter
FRAP: The ferric reducing ability of plasma
PC: Positive correlations
